# City Mortality

**Published:** 1867-01

**Authors:** 


CITY MORTALITY.—The following is the report of the Health-Officer of the mortality of the City of Chicago for the month of December, 1866:

CAUSES OF DEATH.

| | | | |
|---|---|---|---|
| Accidents, | 4 | Fever, Scarlet, | 13 |
| Asthma, | 2 | Fever, Typhoid, | 4 |
| Bronchitis, | 1 | Fever, Typhus, | 1 |
| Burned, | 1 | Fever, not stated, | 1 |
| Cancer, | 2 | Hydrocephelus, | 7 |
| Childbed, | 2 | Inflammation of Kidneys, | 1 |
| Cholera Infantum, | 1 | Inflammation of Bowels, | 9 |
| Consumption, | 42 | Inflammation of Lungs, | 6 |
| Convulsions, | 35 | Inflammation of Liver, | 1 |
| Croup, | 7 | Marasmus, | 1 |
| Cold, | 2 | Old Age, | 15 |
| Chicken Pox, | 1 | Palsy, | 2 |
| Congestion of Brain, | 3 | Pneumonia, | 7 |
| Congestion of Lungs, | 2 | Phthisis Pulmonalis, | 1 |
| Decline, | 1 | Spasms, | 1 |
| Delirium Tremens, | 1 | Spinal Meningitis, | 1 |
| Diarrhœa, | 5 | Suffocation, | 2 |
| Diphtheria, | 15 | Small Pox, | 2 |
| Disease of Heart, | 8 | Suicide, | 1 |
| Disease of Liver, | 2 | Stillborn, | 10 |
| Disease of Lungs, | 7 | Teething, | 3 |
| Disease of Brain, | 6 | Whooping-Cough, | 17 |
| Dropsy, | 7 | Gun-shot Wound, | 1 |
| Drowned, | 2 | White Swelling, | 1 |
| Erysipelas, | 3 | Unknown, | 33 |
| Fever, Childbed, | 2 | | |
| Fever, Remittent, | 4 | Total, | 309 |

AGES OF THE DECEASED.—Under 5 years, 153; over 5 and under 10 years, 19; over 10 and under 20, 15; over 20 and under 30, 28; over 30 and under 40, 34; over 40 and under 50, 16; over 50 and under 60, 11; over 60 and under 70, 11; over 70 and under 80, 12; over 80 and under 90, 6; unknown, 4. Total, 309.

DIVISIONS OF THE CITY.

North, 75 | South, 105 | West, 129 | Total, 309

| | |
|---|---|
| Total number during the month of November, | 382 |
| Decrease from last month, | 73 |
| Total number last year for the month of December, | 333 |

### NATIVITIES.

| | | | | | |
|---|---|---|---|---|---|
| Chicago, | 128 | England, | 5 | Sweden, | 5 |
| Other States, | 63 | Germany, | 47 | Scotland, | 4 |
| Belgium, | 1 | Ireland, | 38 | Wales, | 1 |
| Bohemia, | 2 | Norway, | 6 | Unknown, | 6 |
| Canada, | 2 | On the Sea, | 1 | Total, | 309 |

